# High *In Vitro* and *In Vivo* Activity of BI-847325, a Dual MEK/Aurora Kinase Inhibitor, in Human Solid and Hematologic Cancer Models

**DOI:** 10.1158/2767-9764.CRC-22-0221

**Published:** 2023-10-25

**Authors:** Vincent Vuaroqueaux, Alexandra Musch, Anne-Lise Peille, Gerhard Kelter, Loreen Weichert, Thomas Metz, Hans R. Hendriks, Heinz-Herbert Fiebig

**Affiliations:** 1 4HF Biotec GmbH, Freiburg, Germany.; 2 Charles River, Discovery Research Services GmbH, Freiburg, Germany.; 3 Hendriks Pharmaceutical Consulting, Purmerend, the Netherlands.

## Abstract

BI-847325 is an ATP-competitive inhibitor of MEK/Aurora kinases with the potential to treat a wide range of cancers. In a panel of 294 human tumor cell lines *in vitro*, BI-847325 was found to be a highly selective inhibitor that was active in the submicromolar range. The most sensitive cancer types were acute lymphocytic and myelocytic leukemia, melanomas, bladder, colorectal, and mammary cancers. BI-847325 showed a broader range of activity than the MEK inhibitor GDC-0623. The high efficacy of BI-847325 was associated with but not limited to cell lines with oncogenic mutations in *NRA*S, *BRAF,* and *MAP2K1*.

The high antiproliferative activity of BI-847325 was validated *in vivo* using subcutaneous xenograft models. After oral administration of 80 and 40 mg/kg once weekly for 3 or 4 weeks, BI-847325 was highly active in four of five colorectal, two of two gastric, two of two mammary, and one of one pancreatic cancer models (test/control < 25%), and tumor regressions were observed in five of 11 cancer models. The treatment was well tolerated with no relevant lethality or body weight changes. In combination with capecitabine, BI-847325 displayed synergism over single-agent therapies, leading to complete remission in the triple-negative mammary model MAXFTN 401, partial regression in the colon model CXF 1103, and stasis in the gastric models GXA 3011 and GXA 3023. In conclusion, dual MEK/Aurora kinase inhibition shows remarkable potential for treating multiple types of hematologic and solid tumors. The combination with capecitabine was synergistic in colorectal, gastric, and mammary cancer.

**Significance:**

We report the preclinical evaluation of BI-847325, a MEK/Aurora kinase inhibitor. Our data demonstrate that BI-847325 has potent antitumor activity in a broad range of human solid and hematologic cancer models *in vitro* and *in vivo* and is well tolerated in animal models. It also shows synergistic effect when combined with capecitabine. These findings provide a strong rationale for further development of BI-847325 as a potential therapeutic for patients with cancer.

## Introduction

Over the last three decades, targeted small molecules have emerged as important components of pharmacologic cancer therapies, offering multiple advantages in terms of efficacy and safety compared with traditional chemotherapeutic drugs (1). Among these, kinase inhibitors represent the largest group, with up to 75 FDA-approved drugs (2). The human kinome comprises approximately 535 proteins that play crucial roles in cell growth, proliferation, and differentiation. In many instances, these proteins have been demonstrated to be frequently altered or dysregulated in cancer cells and contribute to tumor development (3).

However, the use of small-molecule kinase inhibitors for cancer therapy remains challenging, as is evident from the low response rates and the emergence of resistance, particularly when therapies are directed against a single target. However, drug combinations overcome some of these issues and display higher efficacy and a reduced risk of relapse. For example, combining the BRAF inhibitor dabrafenib and the MEK inhibitor trametinib leads to long-term clinical benefits in patients with melanoma (4). Consequently, the development of small molecules that inhibit multiple kinases involved in deregulated signal transduction pathways is a promising approach (5).

BI-847325 is an orally bioavailable ATP-competitive inhibitor that targets cancer cells via Aurora and MEK kinases (6). It was discovered in a lead optimization program to develop highly potent Aurora B inhibitors. BI-847325 inhibited the activity of *X. laevi* Aurora kinase B, which is closely related to its human ortholog (92% homology within the kinase domain with no critical difference in the ATP-binding site) with an IC_50_ of 3 nmol/L; the IC_50_ values for human Aurora A and C were 25 and 15 nmol/L, respectively (7, 8). Inhibition of the nuclear kinases Aurora A, B, and C disrupts spindle pole organization, chromosomal segregation, and cell division, thus leading to mitotic catastrophe and cell death (9–11). Inhibition of Aurora B creates typical polyploidization, perturbing cytokinesis, and chromosome orientation by creating misalignment. Multiple inhibitors, such as alisertib targeting Aurora A kinase or barasertib, which inhibits Aurora B kinase, are currently at various stages of clinical development for the treatment of leukemia, lymphoma, and advanced solid malignancy (12, 13). Clinical trials have shown some benefits, mainly in hematologic cancers, and some have also been observed in solid tumors (12, 13). However, the overall clinical response was lower than expected when the drug was administered as a single agent. Therefore, Aurora kinase inhibitors have been studied in various combinations, such as with TORC1/2 inhibitor (TAK-228), paclitaxel, and the VEGFR inhibitor pazopanib (14–16).

In addition to the Aurora kinase inhibitory component, BI-847325 also inhibited MAP2K1 (MEK1) and MAP2K2 (MEK2) in the nanomolar range (IC_50_: 25 and 4 nmol/L, respectively). With their strategic position between RAS-RAF and ERK in the oncogenic MAPK signaling pathway, MEK 1 and 2 are among the most attractive targets for the treatment of a broad range of cancer types (melanoma, thyroid, large part of tumors of the digestive and urogenital systems) carrying *RAF* or *RAS*-activating alterations (17–19). MEK1 and MEK2 inhibitors were discovered 20 years ago (20); however, 18 years were required to see trametinib as the first MEK inhibitor to receive regulatory approval for the treatment of *BRAF V600E*-mutated tumors (16, 21, 22). Most first-generation MEK inhibitors showed only limited efficacy as single agents and failed to demonstrate clinical activity (23, 24). The latest generation of MEK inhibitors has been developed, with special emphasis on the inhibition of *RAS*-mutated tumors (25). Drugs such as selumetinib have shown more favorable profiles in multiple clinical trials (26).

However, the investigations on MEK inhibition revealed that only part of *RAS* or *RAF*-mutant tumors was sensitive and that other parameters inherent to tumor cells determine sensitivity. Activation or lack of inhibition of pathways parallel to the MAPK pathway could limit the efficacy of MEK inhibitors when used as a single agent. The use of MEK inhibitors in combination with other anticancer agents, such as PI3K/AKT, EGFR, mitotic inhibitors, and immunomodulatory drugs, appears to be more promising and is being increasingly studied (27–31).

With both Aurora and MEK inhibitory components, BI-847325 was shown to overcome acquired resistance to BRAF inhibitors in two-dimensional (2D) and three-dimensional *in vitro* settings and in *in vivo* models of melanoma (7). Interest in this compound increased further when preclinical studies demonstrated that the simultaneous blockade of MEK and Aurora kinases by combining the respective inhibitors was superior to single-agent therapy (32–34). In a clinical phase I study, BI-847325 was administered orally once daily for 14 days, followed by a 7-day break in 3-week cycles or once daily for 5 days, followed by a 2 days break, repeated every week (35). The compound had an acceptable safety profile, with some patients experiencing partial response and stable disease. The dose-limiting toxicities were hematologic and gastrointestinal. At the maximum tolerated dose (MTD), no correlation was found between markers of MEK or Aurora kinase inhibition and exposure to BI-847325, resulting in the discontinuation of compound development. Preclinical investigations of BI-847325 contrasted with these results. Thus, the compound was active in the low nanomolar range in both wild-type, and *BRAF-* and *KRAS*-mutated tumor models *in vitro* and *in vivo* (6, 8). BI-847325 demonstrated higher efficacy in xenograft models *in vivo* when the same total dose was administered weekly rather than daily (8). Inhibition of tumor growth has been observed in melanoma and non–small cell lung (NSCL) cancer models *in vivo*. The antitumor effects of the compound were attributed to MEK inhibition in *BRAF*-mutant models and to Aurora kinase inhibition in *KRAS*-mutated models (8).

Although BI-847325 has been shown to be potent in inhibiting tumor models with *RAS*/*RAF*-activating mutations, little is known about the effect of the Aurora kinase inhibitory component of the compound. Preclinical data on BI-847325 remain sparse; hence, the spectrum of tumor types that may benefit from this therapy, particularly those without activating *RAF/RAS* mutations, remains unknown. However, its efficacy and potency across a wide range of tumor types have not yet been reported. As Aurora kinase and MEK inhibition may act synergistically, we hypothesized that BI-847325 could have antitumor activity in a much broader range of tumors than initially anticipated.

Here, we studied the antitumor activity of BI-847325, both alone and in combination with capecitabine, using a broad range of *in vitro* and *in vivo* tumor models. In addition, we used the molecular characteristics of the tumor models to interpret the drug response data.

## Materials and Methods

### Chemicals

BI-847325 and GDC-0623 (Selleckchem) were dissolved in DMSO and diluted in medium for use in *in vitro* experiments. For the *in vivo* experiments, BI-847325 was solubilized in 1% 2-hydroxyethyl cellulose, polysorbate 80, with the pH adjusted to 2.8 with 1 mol/L HCl.

### Tumor Cell Lines

The antiproliferative activity of BI-847325 was investigated *in vitro* using a panel of 294 human tumor cell lines (CL) that were either established in-house or obtained from commercial sources. Details regarding the characteristics of the 294-CL panel, including the culture conditions, resource designation, and molecular data availability, are provided in Supplementary Table S1. All experiments were performed by Charles River Discovery Research Services (DRS) Germany GmbH (RRID:SCR_003792). CLs were authenticated using short tandem repeat analysis at DSMZ and were tested for *Mycoplasma* contamination. The molecular annotation of the tumor models was obtained from previous studies (36, 37) and is available at https://compendium.criver.com/.

### Cell Survival and Proliferation Assay

A modified propidium iodide assay was used to assess the capacity of BI-847325 and GDC-0623 to inhibit the survival and proliferation of CLs grown either in a 2D monolayer culture for cell lines derived from solid tumors or in suspension culture for cell lines derived from hematologic malignancies (37, 38). Briefly, cells were seeded in 96-well plates, and after a recovery period, treated with 10 µL of culture medium or culture medium containing the compound. The drugs were applied at 10 concentrations in half-log increments from 0.001 to 30 µmol/L for 4 days. The cells were then washed with PBS, and a solution containing 7 µg/mL propidium iodide and 0.1% (v/v) Triton X-100 was added. The fluorescence was measured using an EnSpire Multimode Plate Reader (PerkinElmer). Drug effects on cell proliferation and survival were expressed as test/control (T/C) × 100 (%) values. The absolute and relative IC_50_ and IC_70_ values were calculated using a four-parameter nonlinear curve fit (Charles River Data Warehouse Software). Absolute IC_70_ values (Abs IC_70_) were chosen to report their antiproliferative activity *in vitro*.

### *In Vivo* Efficacy in Cell Line–derived and Patient-derived Xenograft Models

The experiments were carried out at the Charles River DRS facilities in Freiburg, Germany, following the recommendations of the Society of Laboratory Animals (GV SOLAS) for the Care and Use of Laboratory Animals, and conducted in accordance with the German Animal Welfare Act. This study was approved by the Committee on Ethics of Animal Experiments of the Regional Council (Regierungspräsidium Freiburg, Abt. Landwirtschaft, Ländlicher Raum, Veterinär- und Lebensmittelwesen, ref. 34, permit no.: G-13/13).

*In vivo* experiments were performed using patient-derived xenografts (PDX) and cell line–derived xenografts (CDX). PDXs were established from surgically resected tumors at Oncotest GmbH (RRID:SCR_000489, since 2015 Charles River Germany DRS GmbH, RRID:SCR_003792), as described previously (39–41). For drug treatments, fragments from serially passaged PDX or 5 × 10^6^ cells from CLs (for CDX) were implanted or injected subcutaneously into the flanks of female nude mice (NMRI nu/nu or SCID, Charles River) at the age of 6–8 weeks. Each tumor model was implanted bilaterally in 2 mice per group (four tumors) in the screening series and 3 mice (six tumors) in the combination studies. Tumor growth was assessed using caliper measurements of each tumor in two perpendicular diameters, and volumes were calculated according to the formula *a* × *b*^2^/2, where *a* is the longest diameter and *b* is the perpendicular axis. When the tumors reached a volume between 50 and 150 mm^3^, the mice were randomized into groups based on tumor volume and body weight. Tumor growth and body weight were measured twice per week. Vehicle (1% 2-hydroxyethyl cellulose, 0.25% polysorbate 80, pH 2.8), BI-847325, or GDC-0623 (40 or 80 mg/kg/day) were administered orally once a week for 3 or 4 weeks or daily for 2 weeks within 24 hours after randomization. Mice were sacrificed when the tumors reached a volume of 1,800 mm^3^. Capecitabine was administered orally once daily at either 200 or 150 mg/kg/day (days 1–7). In combination, capecitabine was administered at the same dose and scheduled as in the monotherapy, and BI-847325 was injected one hour later either simultaneously (days 1, 8, and 15) or sequentially (days 8, 15, and 22) at 80 mg/kg/day. Tumor growth inhibition was determined on each day of measurement by calculating the ratio of the median relative tumor volume values of the test versus the control group multiplied by 100 (T/C %). The optimal (minimum) value was used to evaluate the antitumor activity of the test compounds. In addition, the median absolute and relative tumor volumes were calculated. The criteria for activity and synergism are shown in Figs. 4 and 5.

*In vivo* antitumor activity of BI-847325 was defined as followed: +, moderate activity, 25% ≤ T/C <50%; ++, high activity, 10% ≤T/C<25%; +++, very high activity, 5% ≤ T/C<10%. The term “tumor stasis” refers to a relative tumor volume between 75% and 125% of the initial volume after treatment. The term “partial tumor regression” refers to a relative tumor volume between >0% and 75%.

### Statistical Analysis

Statistical analyses were carried out using GraphPad Prism version 9.0 (RRID:SCR_002798) or in “R” statistical computing environment (R statistical software version 3.4.4; http://www.R-project.org/, RRID:SCR_001905; ref. 42). For all tests in the study, P values less than 0.05 were considered statistically significant.

### Data Availability

The data generated in this study are available in the article and Supplementary Data.

## Results

### Potency and Selectivity of BI-847325-mediated Inhibition in 2D Cell-based Assay *In Vitro*

**FIGURE 1:**
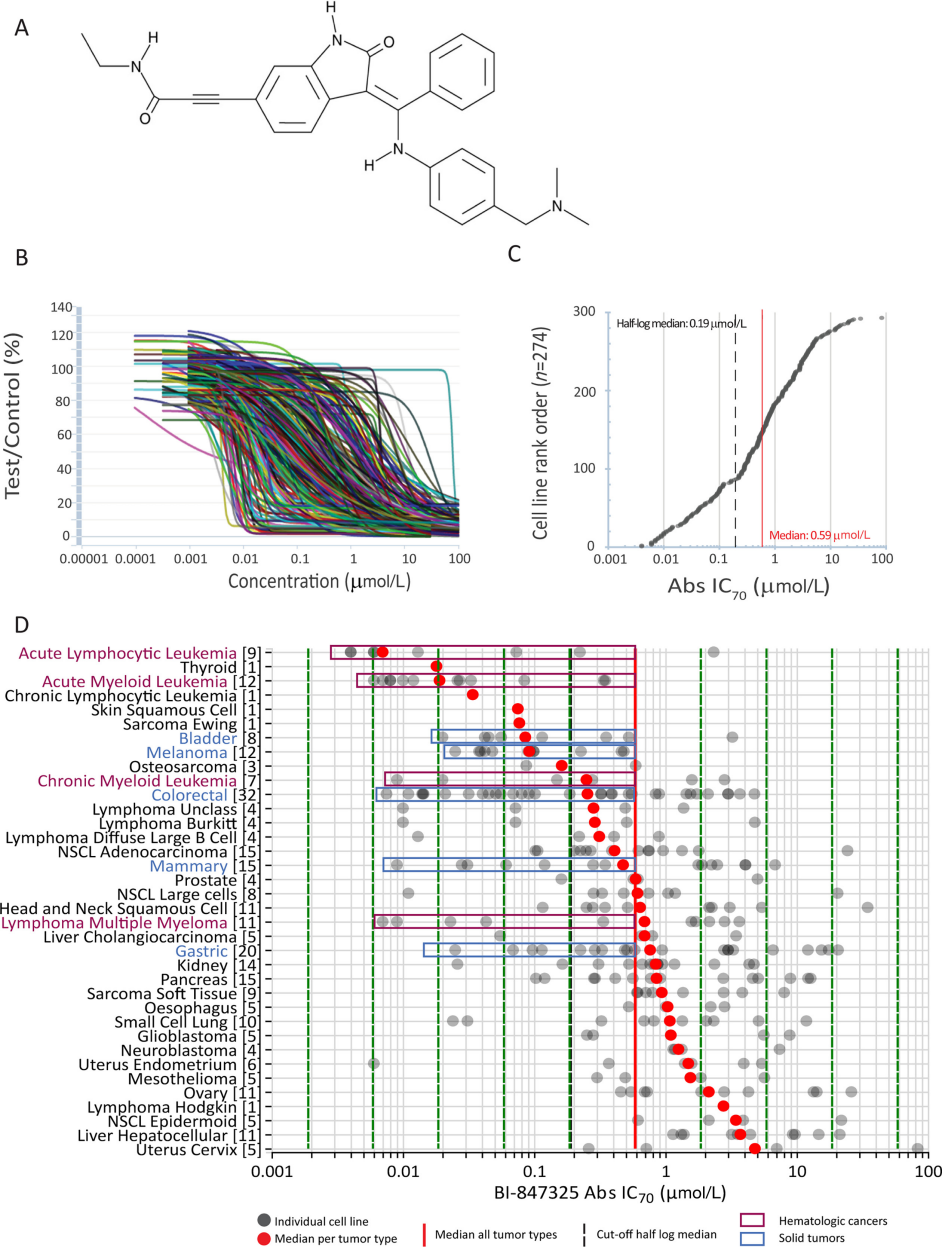
*In vitro* antiproliferative activity of BI-847325 tested in 294 cancer CLs. **A,** BI-847325 Chemical structure. **B,** individual dose–response curves of BI-847325 in 294 solid tumor and hematologic cancer CLs after 4 days of continuous exposure. Horizontal axis: BI-847325 concentration in µmol/L (log scale). Vertical axis: drug effect on cell survival, expressed as percentage of growth inhibition in BI-847325–treated cells compared with the growth of vehicle-treated control cells [T/C × 100 (%)]. **C,** BI-847325 sensitivity rank order for all CLs. The red line represents the median Abs IC_70_ value across all CLs (0.59 µmol/L). The dashed black line represents the half-log median (median/10^0.5^) which is used as a cutoff to discriminate between highly sensitive CLs (<0.19 µmol/L) and less sensitive to strongly resistant CLs (≥0.19 µmol/L). **D,** Scatter plot showing the *in vitro* antiproliferative activity of BI-847325 across 36 CL cancer types. Horizontal axis: Abs IC_70_ value per CL; vertical axis: tumor types sorted from the top to the bottom by increasing median IC_70_ values. The total number of CLs per tumor type is shown in parentheses. The red dots represent the median Abs IC_70_ values for each tumor type. The solid vertical red line represents the overall median Abs IC_70_ of all CLs. The dashed black line indicates the cut-off value at 0.19 µmol/L separating the BI-847325 sensitivity subsets. The colored rectangles highlight tumor types with more than three sensitive CLs (<0.19 µmol/L); dark blue: CLs derived from solid tumors; dark red: CLs from hematologic cancers.

BI-847325 [formula:3-[(3E)-3-[[4-[(dimethylamino)methyl]anilino]-phenylmethylidene]-2-oxo-1H-indol-6-yl]-N-ethylprop-2-ynamide (C29H28N4O)] (Fig. 1A) is an orally bioavailable ATP-competitive inhibitor of Aurora and MEK kinases. First, we investigated the antiproliferative activity of the compound *in vitro* in a panel of 294 human tumor cell lines, covering 27 histologic types of solid tumors, four leukemia, and five lymphoma types (Table 1; Supplementary Table S1).

**TABLE 1:** Human cell line panel (*n* = 294) and cancer types investigated for BI-847325 efficacy

Tumor types	No. of CLs	%
**CLs derived from solid tumors**		
Bladder	8	2.7%
Mammary	15	5.1%
Colorectal	32	10.9%
Head and neck squamous cell	11	3.7%
Liver cholangiocarcinoma	5	1.7%
Liver hepatocellular	11	3.7%
Melanoma	12	4.1%
Mesothelioma	5	1.7%
Glioblastoma	5	1.7%
Neuroblastoma	4	1.4%
NSCL large cell	8	2.7%
NSCL adenocarcinoma	15	5.1%
NSCL epidermoid	5	1.7%
Oesophagus	5	1.7%
Ovary	11	3.7%
Pancreas	15	5.1%
Prostate	4	1.4%
Kidney	14	4.8%
Sarcoma Ewing	1	0.3%
Sarcoma soft tissue	9	3.1%
Osteosarcoma	3	1.0%
Skin squamous cell	1	0.3%
Small cell lung	10	3.4%
Gastric	20	6.8%
Thyroid	1	0.3%
Uterus cervix	5	1.7%
Uterus endometrium	6	2.0%
**CLs derived from hematologic cancers**		
Acute lymphocytic leukemia	9	3.1%
Acute myeloid leukemia	12	4.1%
Chronic lymphocytic leukemia	1	0.3%
Chronic myeloid leukemia	7	2.4%
Lymphoma Burkitt	4	1.4%
Lymphoma diffuse large B cell	4	1.4%
Lymphoma Hodgkin	1	0.3%
Lymphoma multiple myeloma	11	3.7%
Lymphoma unclass	4	1.4%
Total	294	100.0%

As depicted in Fig. 1B, a wide range of dose–response curves and sensitivities to BI-847325 were obtained. BI-847325 exhibited a strong potency with absolute IC_70_ values ranging from 0.004 to > 30 µmol/L and a median Abs IC_70_ value of 0.59 µmol/L [interquartile range (IQR): 0.1–2.24 µmol/L; Fig. 1C]. The efficacy of BI-847325 was submicromolar in 183 of the 294 CLs (62%). A total of 87 CLs with Abs IC_70_ values below the half-log of the median value (half-log median Abs IC_70_ = 0.19 µmol/L) were highly sensitive.

BI-847325 demonstrated pronounced selectivity, with significant differences in sensitivity within and across tumor entities (Kruskal–Wallis, *P* < 0.0001; Fig. 1D; Supplementary Table S2). CLs highly sensitive to BI-847325 (Abs IC_70_ < 0.19 µmol/L) were identified in most tested tumor types. Focusing on the tumor types for which more than five CLs were investigated, and more than 20% of CLs were highly sensitive to BI-847325 (Abs IC_70_ < 0.19 µmol/L), acute lymphocytic leukemia (78%), acute myeloid leukemia (AML, 83%), chronic myeloid leukemia (CML, 43%), and multiple myeloma lymphoma (36%) were the most sensitive hematologic cancer types. On average, CLs from bladder (63%), melanoma (75%), colorectal cancer (50%), mammary (33%), gastric (20%), and small cell lung (20%) cancers were the most sensitive solid tumor entities (Supplementary Table S3).

### BI-847325 Comparison with the MEK Inhibitor GDC-0623

**FIGURE 2:**
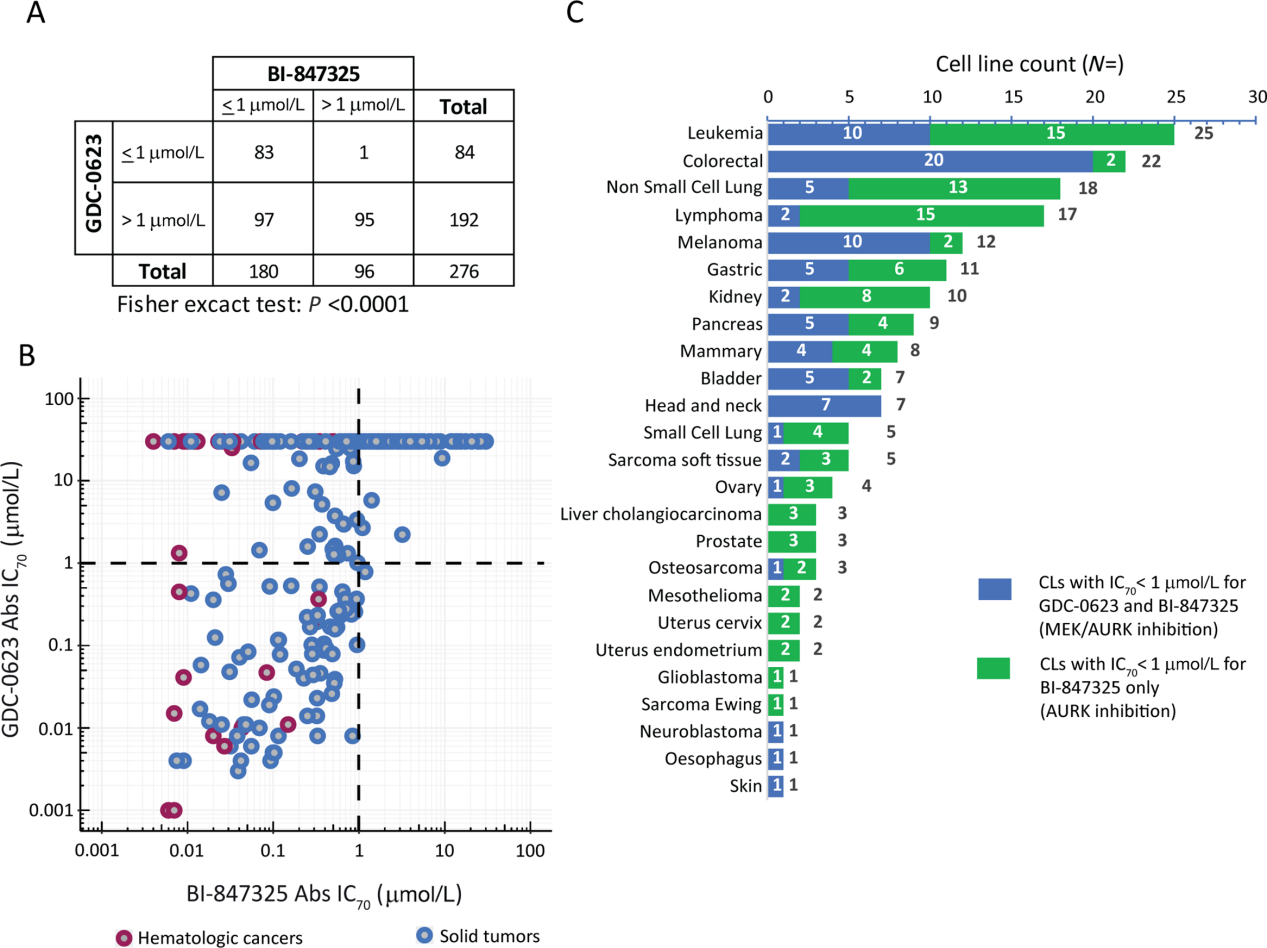
Comparison between the *in vitro* antiproliferative activities of BI-847325 and the MEK inhibitor GDC-0623. **A,** Contingency table with counts of CLs per class of sensitivity to BI-847325 and GDC-0623 (cutoff for Abs IC_70_ values for both compounds: ≤ 1 µmol/L; statistics: Fisher exact test, significance: *P* < 0.05). **B,** Correlation plot of BI-847325 and GDC-0623 Abs IC_70_ values for 276 CLs. Dark blue circles: CLs derived from solid tumors, Dark red circles: CLs derived from hematologic cancers. **C,** Counts of CLs per tumor type being sensitive to GDC-0623 and/or BI-847325 (Abs IC_70_ ≤ 1µmol/L). Dark blue bars: CLs sensitive to both GDC-0623 and BI-847325, Green bars: CLs sensitive to BI-847325 only. The total number of CLs per tumor type is indicated in black on the right of each bar.

We compared the antitumor profile of BI-847325 with that of GDC-0623, which was concomitantly tested with BI-847325 in 276 CLs (Fig. 2).

Like BI-847325, GDC-0623 was selective and preferentially active in CLs derived from melanoma, colorectal, chronic myeloid leukemia (CML), bladder, and acute myeloid leukemia (AML) (Supplementary Fig. S1). However, the GDC-0623 was clearly less potent than BI-847325 (median Abs IC_70_ value = 30 versus 0.59 µmol/L, IQR: 0.26–30 vs. IQR: 0.1–2.24 µmol/L, respectively). Using an IC_70_ of 1 µmol/L as a cutoff for the comparison of the activity of both compounds, we observed significant differences in the number of CLs sensitive to BI-847325 and GDC-0623 (Fisher exact test: *P* < 0.0001; Fig. 2A; Supplementary Table S4). The CL panel contained more than twice as many CLs sensitive to BI-847325 (*N* = 180) than to GDC-0623 (*N* = 84). Two distinct subsets of CLs were identified by plotting the IC_70_ data for both compounds (Fig. 2B). One subset (*N* = 179) showed a positive correlation between the Abs IC_70_ values of both compounds (Spearman ϼ = 0.85, *P* < 0.0001; Supplementary Table S4). The second subset of CLs (*N* = 97) was sensitive to BI-847325 (Abs IC_70_ ≤ 1 µmol/L) but showed resistance to GDC-0623 (Abs IC_70_ > 1 µmol/L). Compared with GDC-0623, BI-847325 extended the number of CLs inhibited in the submicromolar range in most of the tumor entities investigated (Fig. 2C). This was observed for lymphoma (*N* = 15), leukemia (*N* = 14), and NSCL (*N* = 13) tumor types, as well as in various other solid tumor entities, such as gastric, pancreatic, or kidney cancer-derived CLs.

### Impact of *RAF/RAS/MAPK* Oncogenic Alterations on CLs Sensitivity to BI-847325 *In Vitro*

**FIGURE 3:**
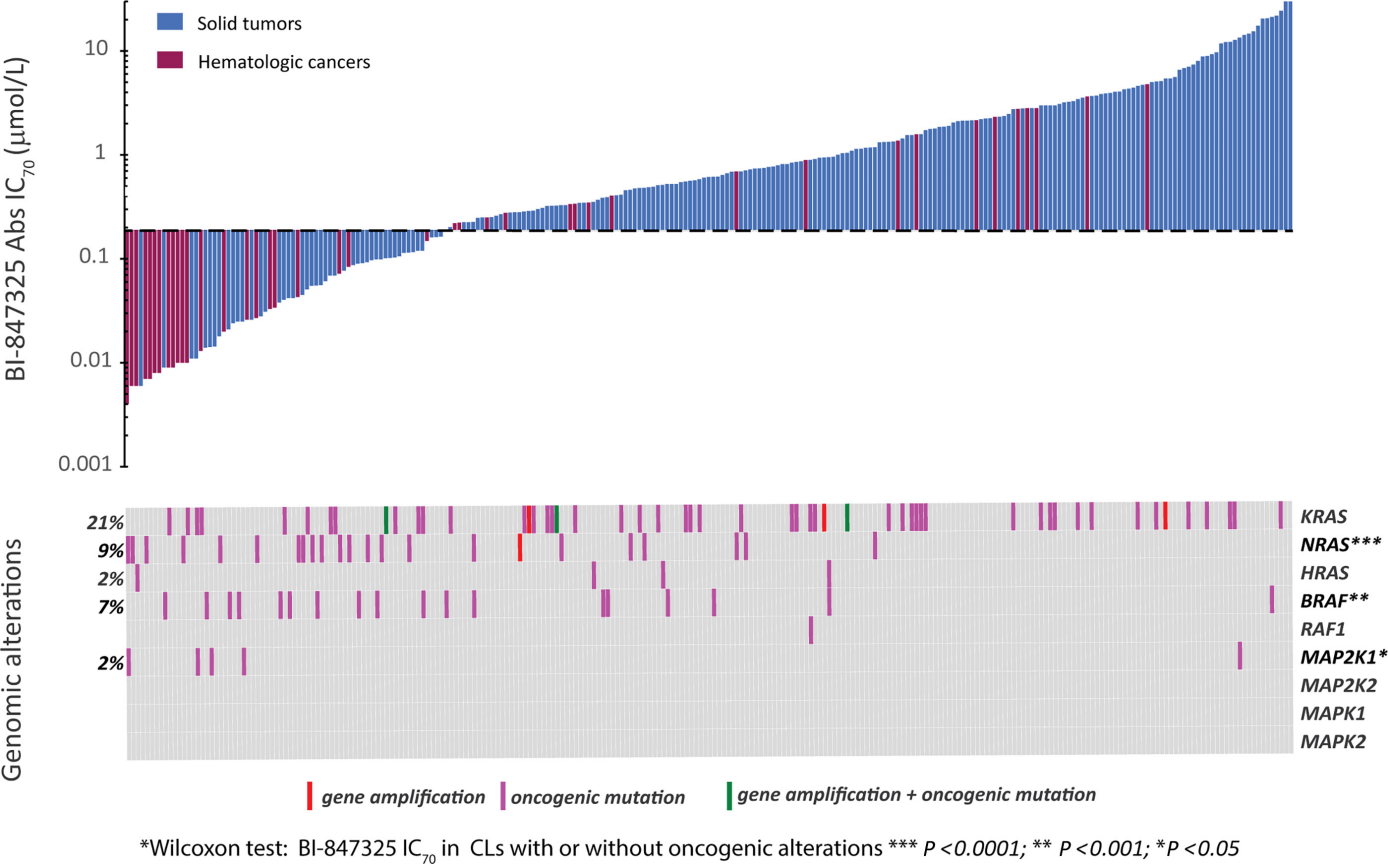
Association between cell line sensitivities to BI-847325 *in vitro* and their oncogenic alterations in the RAS/RAF/MAPK pathway. Top: Waterfall plot of BI-847325 Abs IC_70_ values (*y*-axis) per cell line (*x*-axis). The dashed black line separates the CLs that are sensitive (Abs IC_70_ < 0.19 µmol/L) from those that are less sensitive (Abs IC_70_ ≥ 0.19 µmol/L) to BI-847325. Dark blue: CLs derived from solid tumors; dark red: CLs from hematologic cancers. Bottom: Genomic alterations (gene amplifications and/or oncogenic mutations) in the MAPK pathway for each of the 253 CLs, characterized by whole-exome sequencing and Affymetrix genome-wide human SNP array 6.0 (SNP6.0). The percentage of CLs being altered for each gene is indicated on the left of the plot. The statistical significance of the association between the genomic alterations and the BI-847325 Abs IC_70_ values is indicated by asterisks on the right of the gene symbols.

We investigated whether alterations in the genes encoding components of the MAPK signal transduction pathway contributed to cell sensitivity to BI-847325 (Fig. 3). The annotation of these genes was available for 253 CLs and included exome mutations, gene amplifications, and gene deletions. Our CL panel showed oncogenic alterations in *KRAS* (21%), *NRA*S (9%), *HRAS* (2%), *BRAF* (7%), and *MAP2K1* (2%). A single CL had a *RAF1* mutation*,* and no alterations were found in *MAP2K2*, *MAPK1,* and *MAPK2* genes (Supplementary Table S5). Statistical analysis demonstrated that oncogenic alterations in *NRAS*, *BRAF*, and *MAP2K1* were significantly associated with sensitivity to BI-847325 (Wilcoxon test, *P* < 0.05). CLs carrying *BRAF* V600E/M, R354Q, and D211G mutations and *NRAS*-activating mutations Q61L, K, and R or, less frequently, G12C, D, and G13D displayed the highest sensitivity to BI-847325 (Supplementary Table S5). *BRAF* mutations are prevalent in CLs derived from melanoma and colorectal cancers, and *NRAS* in melanoma, leukemia, bladder cancer, and lymphoma. Remarkably, four of the five CLs with *MAP2K1*-activating mutations (Q56P, D67N, and Y134C) were sensitive to BI-847325. *KRAS* alterations were not significantly associated with CL sensitivity to BI-847325. A subset of *KRAS*-mutated CLs, mainly derived from hematologic and gastrointestinal cancers, was sensitive to BI-847325.

### Efficacy of BI-847325 in Subcutaneous Tumor Xenograft in Mice

*In vivo* experiments were performed to investigate the antitumor activity of BI-847325 in mice bearing 11 subcutaneously implanted PDX or CDX models. The cancer models were selected on the basis of tumor type, mutation status, and *in vitro* sensitivity (either high or intermediate sensitivity) and included five colorectal (CXF 1103, CXF 260, RKO, HCT-116, COLO 205), two gastric (GXA 3011, GXA 3023), two mammary (MAXFTN 401, MDA-MB-231), bladder RT112, and pancreatic MIA-PaCa-2. MIA-PaCa-2 was a positive control based on its known sensitivity to BI-847325 *in vivo* (8). *BRAF* mutations were present in three models: V600E in colon cancer RKO, COLO 205, and G464V in mammary cancer MDA-MB-231 cells. Five models had *KRAS* mutations: G13D in GXA 3023, HCT-116, and MDA-MB-231; G13R in CXF 260; and G12C in MIA-PaCa-2. None of the models carried mutations in *MAP2K1* and *NRAS* genes.

Eight of the 11 models were tested at two dose levels of BI-847325 [40 and 80 mg/kg, orally, once per week for either 4 (seven models) or 2 weeks (one bladder cancer model)], and three colon cancer models (COLO 205, HCT-116, CXF 260) were tested only at the highest BI-847325 dose level for 3 weeks. The experiments consisted of a vehicle control group in addition to the BI-847325–treated groups and were performed using an *in vivo* screening format, with 2 mice per group bearing bilateral subcutaneous tumors of the same PDX or CDX model per mouse, that is, four tumors per dose group (*n* = 4). In the experiments with COLO 205, HCT-116, and CXF 260, the group size was three mice.

**FIGURE 4:**
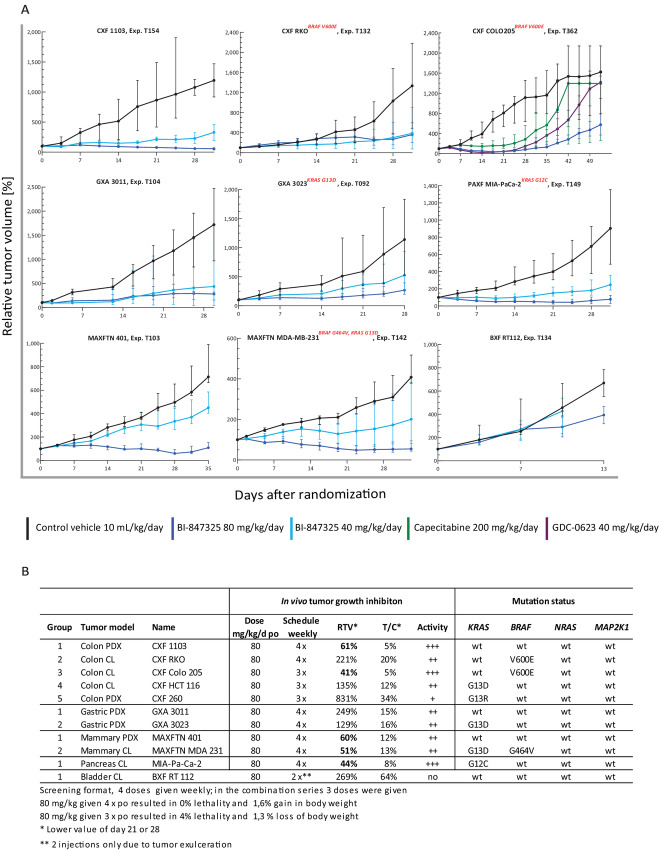
*In vivo* antitumor growth efficacy of BI-847325 in 11 solid tumor models grown as subcutaneous xenografts in nude mice. **A,** Tumor growth curves over time are shown as the relative tumor volumes (%). BI-847325 (either at 80 or 40 mg/kg/dose), and the vehicle were administered orally once per week for 3 or 4 weeks. Black curves: vehicle control at 10 mL/kg/dose; light blue: BI-847325 at 40 mg/kg/day and dark blue: BI-847325 at 80 mg/kg/day; green curve: capecitabine administered orally at 200 mg/kg/day on days 1–7; violet curve: GDC-0623 administered orally at 40 mg/kg/day on days 1–14. CXF 1103 and RKO: colorectal cancer; GXA 3011 and GXA 3023: gastric cancer; MAXFTN 401 and MDA-MB-231: triple-negative mammary cancer; MIA-PaCa-2: pancreatic cancer; RT112: bladder cancer. **B,** antitumor activity of BI-847325 in human tumor models tested at a dose level below the MTD, *in vivo* tumor growth inhibition, and *KRAS*, *BRAF*, *NRAS*, *MAP2K1* mutational status are shown. Rating of BI-847325 antitumor activity is defined in the Materials and Methods section.

The overall outcome of the *in vivo* experiments was highly concordant with the *in vitro* results described above. Overall, the models tested were sensitive to BI-847325 with responses ranging from partial tumor regression to reduced growth rate (Fig. 4A and B). Tumor responses were transient, as in all analyzed cases, the tumor growth resumed or accelerated after the end of the dosing phase. At doses of 40 and 80 mg/kg, BI-847325 exhibited clear dose-dependent antitumor activity in all tumor models, except for the bladder cancer model RT112 (Fig. 4A). The experiment could only be properly analyzed for 7 or 11 days, as the mice had to be sacrificed because of tumor exulceration. At that point, unlike all other models, there was no indication of tumor response to BI-847325.

At 80 mg/kg/week, BI-847325 showed a very high activity in three models with T/C values <10% and a high activity in six out of 11 models with T/C values between 10% and 5%. the most sensitive models were colorectal cancers with T/C values ranging from 5% to 34%, followed by pancreatic cancer with a T/C value of 8%, triple-negative mammary cancers with T/C values of 12% and 13%, and gastric cancers with T/C values of 15% and 16% (Fig. 4B). BI-847325 induced tumor regressions in five of the 11 tested tumor models: two colon cancer models, CXF 1103 and COLO 205, two triple-negative mammary cancer models MAXFTN 401 and MDA-MB-231, and the pancreatic cancer model MIA-PaCa-2. BI-847325 was well tolerated as a single agent. There was only one treatment-related death among the 23 mice that received three or four weekly doses of BI-847325 (80 mg/kg orally). The maximum median group body weight loss was 1.3% for three injections and a gain of 1.6% for four injections with zero lethality (Supplementary Fig. S2). In all cases, the mice regained weight despite the continued dosing.

### Efficacy of BI-847325, MEK Inhibitor GDC-0623, and Capecitabine in Xenograft COLO 205 *In Vivo*

The efficacy of BI-847325 was compared to that of the MEK inhibitor GDC-0623 and the standard-of-care capecitabine in inhibiting the *BRAF* V600E-mutated model COLO 205 (Fig. 4A – top right growth curves). BI-847325, administered at a dose of 80 mg/kg/day (days 1, 8, and 15), and GDC-0623, administered at an MTD of 40 mg/kg/day (days 1–14), showed similar activity with regressions during the treatment period. In contrast, capecitabine at 200 mg/kg/day (days 1–7) effected tumor stasis only. For all three compounds, a tumor regrowth was observed after the last dose of treatment; however, regrowth occurred later with BI-847325 compared with GDC-0623 and capecitabine treatments.

### BI-847325 Shows Synergistic Activity in Combination with the Standard-of-Care Drug Capecitabine in all Four Models of Colon, Gastric, and Triple-negative Mammary Cancer

**FIGURE 5:**
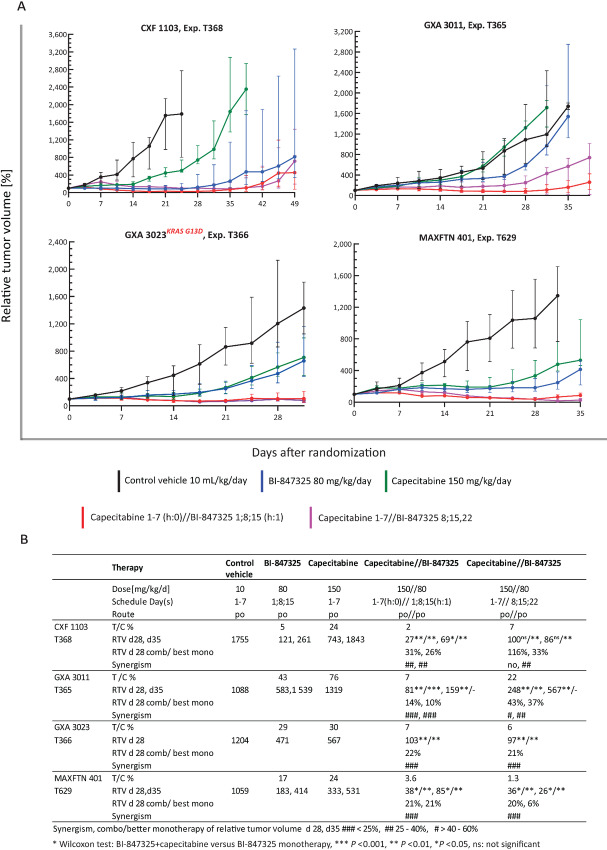
*In vivo* antitumor growth efficacy of BI-847325 in combination with capecitabine. Combinations (simultaneously or sequentially) were compared with both monotherapies. **A,** Tumor growth curves are represented as the relative tumor volume (%) over time (days). Black curves: control vehicle 10 mL/kg/day on days 1–7. Blue curves: BI-847325 administered at 80 mg/kg/day on days 1, 8, and 15. Green curves: capecitabine 150 mg/kg/day on days 1–7. Red curves: BI-847325 administered at 80 mg/kg/day on days 1, 8, and 15 (h:1), plus capecitabine administered at 150 mg/kg/day on days 1–7 (h:0). Purple curves: capecitabine 150 mg/kg/day on day 1–7 plus BI-847325 80 mg/kg/day on days 8, 15, and 22. CXF 1103, colorectal cancer; GXA 3011 and GXA 3023, gastric cancer; MAXFTN 401, triple-negative mammary cancer. **B,** T/C values (%), relative tumor volume (RTV, %), and synergism index based on RTV. Rating of BI-847325 antitumor activity is defined in the Materials and Methods section.

Finally, we evaluated whether the combination of BI-847325 with capecitabine, a prodrug of the antimetabolite 5-fluorouracil, which is the standard of care for mammary, colon, and gastric cancers, could be beneficial for the treatment of these tumor entities. To assess the therapeutic interaction between these two compounds, we chose the following four PDX models: triple-negative mammary cancer MAXFTN 401, colon cancer CXF 1103, and two gastric cancers, GXA 3011 and GXA 3023. In these experiments, BI-847325 was administered at 80 mg/kg orally once a week for 3 weeks, and capecitabine was administered at 150 mg/kg/day orally for 7 consecutive days. The experiments consisted of the following five groups (group size: three mice bearing two tumors each): a vehicle control group; one group administered either BI-847325 or capecitabine monotherapy; and two groups administered BI-847325 and capecitabine in combination, either simultaneously (BI-847325 on days 1, 8, 15, and capecitabine on days 1–7) or sequentially (capecitabine on days 1–7 and BI-847325 on days 8, 15, and 22). Figure 5A and B show that the combination therapy was more effective than the best single-agent therapy in all four models administered either simultaneously or sequentially. The T/C values were between 1.3% and 22% for the combinations and were markedly lower than those of the individual agents (Fig. 5B).

The combination affected a partial tumor regression in three models and proved superior to both corresponding monotherapies. For example, although GXA 3011 was resistant to capecitabine (T/C = 76%) and displayed a reduced growth rate in response to BI-847325 (T/C = 43%), it underwent partial tumor regression with combination therapy (T/C = 7%). While GXA 3023 displayed reduced growth rates under monotherapies (T/C = 29% and 30% for BI-847325 and capecitabine, respectively), combination therapy induced partial tumor regression (T/C = 7% for simultaneous dosing and 6% for sequential dosing). Similarly, MAXFTN 401 exhibited reduced growth rates in response to monotherapies (T/C = 17% and 24% for BI-847325 and capecitabine, respectively) and underwent partial tumor regression following simultaneous (T/C = 3.6%) and sequential (1.3%) combination therapies. The efficacy advantage of the combination was less marked for the CXF 1103 model, which was already highly sensitive to BI-847325 monotherapy. BI-847325 induced partial tumor regression (T/C = 5%) and capecitabine reduced the growth rate (T/C = 24%) in this model. This combination achieved a T/C ratio of 2% after simultaneous dosing. The advantage of the simultaneously administered combination over BI-847325 or capecitabine monotherapy was significant; on days 28 and 35, tumors in the combination group displayed smaller relative volumes than all tumors in the monotherapy group (Wilcoxon test *P* < 0.05).

The increase in the efficacy of the combination therapy was accompanied by a slight increase in body weight loss. The average median body weight loss was for the combinations after simultaneous treatment 9%, range: 5%–13% and after sequential treatment 5.2%, range: 3.0%–7.8%) compared with the monotherapies of BI-847325 with 3.2%, range: 1.7%–5.6%; and capecitabine 2.4%, range: 0%–3.2% (Supplementary Fig. S3). While all mice survived after either capecitabine monotherapy or both agents combined sequentially, one of the 12 mice treated with BI-847325 monotherapy and the simultaneous combination died.

## Discussion

In this study, we explored the antitumor potential of the dual MEK/Aurora kinase inhibitor BI-847325. Dual inhibitors are an emerging class of compounds for cancer treatment that aim to improve efficacy, decrease the risk of relapse, and avoid drug resistance observed when applying single targeting agents. Extended *in vitro* and *in vivo* experiments in a broad range of tumor entities have highlighted the efficacy of this compound, validated its mechanism of action, and revealed the most relevant tumor types for treatment. It also demonstrated robust and synergistic activity when administered in combination with the standard-of-care drug capecitabine. Capecitabine is one of the drugs used as a first-line treatment in metastatic colorectal cancer, in combination with irinotecan, oxaliplatin, or the targeted agents bevacizumab and EGFR inhibitors (43, 44). In metastatic breast cancers, capecitabine is used for later lines of therapy (45), in advanced gastric cancer, in combination with platinum-based chemotherapy and/or immunotherapy and/or Her2-directed agents, depending on molecular subtypes (46).

To the best of our knowledge, this is the first large *in vitro* study to report the antiproliferative effects of BI-847325 in human tumor cell lines with distinct origins and genetic backgrounds. While it does not encompass all the diversities and complexities of cancers, this initial layer of analysis on well-characterized models provides refined information on the types of cancer and genetic alterations that determine cancer cells’ sensitivity and resistance to the compounds. BI-847325 showed a 7,500-fold range of efficacy, from low nanomolar to micromolar concentrations. Sixty-two percent of the CLs had an Abs IC_70_ value below 1 µmol/L and 25% below 0.1 µmol/L. In addition to the already identified sensitive tumor types, such as melanoma and colorectal cancers (7, 8), our study showed that BI-847325 is also highly active in leukemia, lymphoma, bladder, gastric, and some mammary cancers.

Furthermore, our study showed that the high antitumor efficacy of BI-847325 is not restricted to CLs with *NRAS/BRAF/MAP2K1*-activating mutations. The head-to-head comparison of BI-847325 and GDC-0623 highlighted that approximately 35% of CLs in our panel were sensitive to BI-847325, but strongly resistant to MEK inhibition by GDC-0623, suggesting that BI-847325 could increase the proportion of successfully treated tumors over the use of a classical MEK inhibitor. This subset predominantly comprises CLs from blood cancers but also from lung, colorectal, mammary, and other types of solid cancers. All were tumor entities investigated in clinical trials testing Aurora kinase inhibitors (12, 13). As expected, these CLs were mainly wild type for *BRAF*, *NRAS,* or *MAP2K1*; some were *KRAS* mutated, whereas others were not, suggesting that their inhibition was mainly driven by Aurora kinases. Sini and colleagues (8) reported observations similar to those of *in vivo* studies in which BI-847325 was administered once a week at 70 mg/kg. The authors suggested that tumor inhibition was driven by MEK in *BRAF*-mutant models and by Aurora kinases in models mutated for *KRAS*.

We next investigated BI-847325 *in vivo* by using cell lines and patient tumor-derived xenografts models. To investigate the compound in a wide range of models, we first took the approach of a mini-screen format by testing 11 tumor models with bilateral implementations in 2 mice each. While results reliability could be affected, we validated the accuracy of our approach by observing an almost perfect match between the antiproliferative effects of BI-847325 *in vitro* and the corresponding *in vivo* antitumor growth.

Additionally to previous *in vivo* studies which were mainly conducted with melanoma and NSCL cancer tumor models at daily treatment doses of 10 or 20 mg/kg (7, 8), our study showed that, at weekly doses of 40 and 80 mg/kg, BI-847325 demonstrated strong antitumor growth in other cancer types with high clinical needs, such as triple-negative mammary, colorectal, gastric, and pancreatic cancer models. No increased mice lethality was observed, and the limited loss of body weight suggests that the MTD was not reached. These results of BI-847325 tolerability strongly support those reported by Sini and colleagues (8).

In four models of tumor xenografts, the combination of BI-847325 with the antimetabolite capecitabine was beneficial compared with treatment with a single agent. A similar synergism was reported with a combination of Aurora kinase inhibitors and the microtubule inhibitor vincristine in hematologic cancers *in vivo* (47, 48). The strategy of blocking cell cycle in S and M phases with capecitabine and compounds such as vinorelbine showed already manageable safety profile in triple-negative breast cancer (49). With a similar strategy using capecitabine and BI-847325 combination, the same lethality of 8% and the slightly higher body weight loss compared with both as monotherapy seems to be acceptable in view of the strong therapeutic synergism observed in all four models studied. In general, when the same dose of the single-agent therapies is given in the combination, a dose reduction is necessary, which was not needed for BI-847325 plus capecitabine. This warrants further investigation to determine the optimal dosage and to investigate possible adverse effects more thoroughly.

Together, these results suggest that BI-847325 can be combined with other agents to achieve optimal tumor inhibition.

In summary, BI-847325 is a potent and selective inhibitor of the MEK and Aurora kinases. Its dual mechanism of action broadens the array of clinically suitable tumor types compared with MEK or Aurora kinase inhibitors alone. Combination therapy of BI-847325 with capecitabine showed a synergistic effect in all four preclinical models of colon, gastric, and mammary cancers studied suggesting clinical studies in these tumor types. Further detailed biomarker analysis will be needed to refine determinants of cancer cell sensitivity to the compound.

## Supplementary Material

Supplementary Tables 1-5 Five supplementary tables 1 to 5 giving more information about:
- the culture conditions and molecular profile of the 294 cell lines tested.
- the IC70 range per cancer type.
- the number of "sensitive" and "less sensitive to resistant" cell lines per cancer type.
- the IC70 values of BI-847325 and GDC-0623 per cell line.
- the genomic alterations of MAPK pathway-related genes in the cell lines sensitive to BI-847325.

Supplementary Figure S1 Supplementary Figure S1 shows a scatter plot of the GDC-0623 IC70 values across cell lines sorted by cancer entities.

Supplementary Figure S2 Supplementary Figure S2 shows the relative body weights of the mice treated with BI-847325 as monotherapy (first in vivo experiment).

Supplementary Figure S3 Supplementary Figure S3 shows the relative body weights of the mice treated with BI-847325 or capecitabine as monotherapy and in combination (second in vivo experiment).
