# Supplementary material for: High In Vitro and In Vivo Activity of BI-847325, a Dual MEK/Aurora Kinase Inhibitor, in Human Solid and Hematologic Cancer Models
Source: Cancer Res Commun. 2023 Oct 25;3(10):2170–81. doi: 10.1158/2767-9764.CRC-22-0221 (PMC10599287; doi:10.1158/2767-9764.CRC-22-0221)
Supplement: Supplementary Figure S1 — shows a scatter plot of the GDC-0623 IC70 values across cell lines sorted by cancer entities. [file crc-22-0221-s02.pdf]

Supplementary materials

Supplementary Figure S1. Scatter plot showing in vitro antiproliferative activity of the MEK inhibitor GDC-0623 across CLs of 36 cancer types.

Horizontal axis: Abs IC_{70} value per CL; vertical axis: tumor types sorted from the top to the bottom by increasing median Abs IC_{70} values. The total number of CLs per tumor type is shown in parentheses. Red dots represent median Abs IC_{70} values per tumor type. The solid vertical red line represents the overall median Abs IC_{70} of all CLs. Colored rectangles highlight tumor types with more than three CLs displaying GDC-0623 Abs IC_{70} below the BI-847325 cut-off (0.19 µM). Dark blue: CLs derived from solid tumors; dark red: CLs derived from hematologic cancer cell lines.

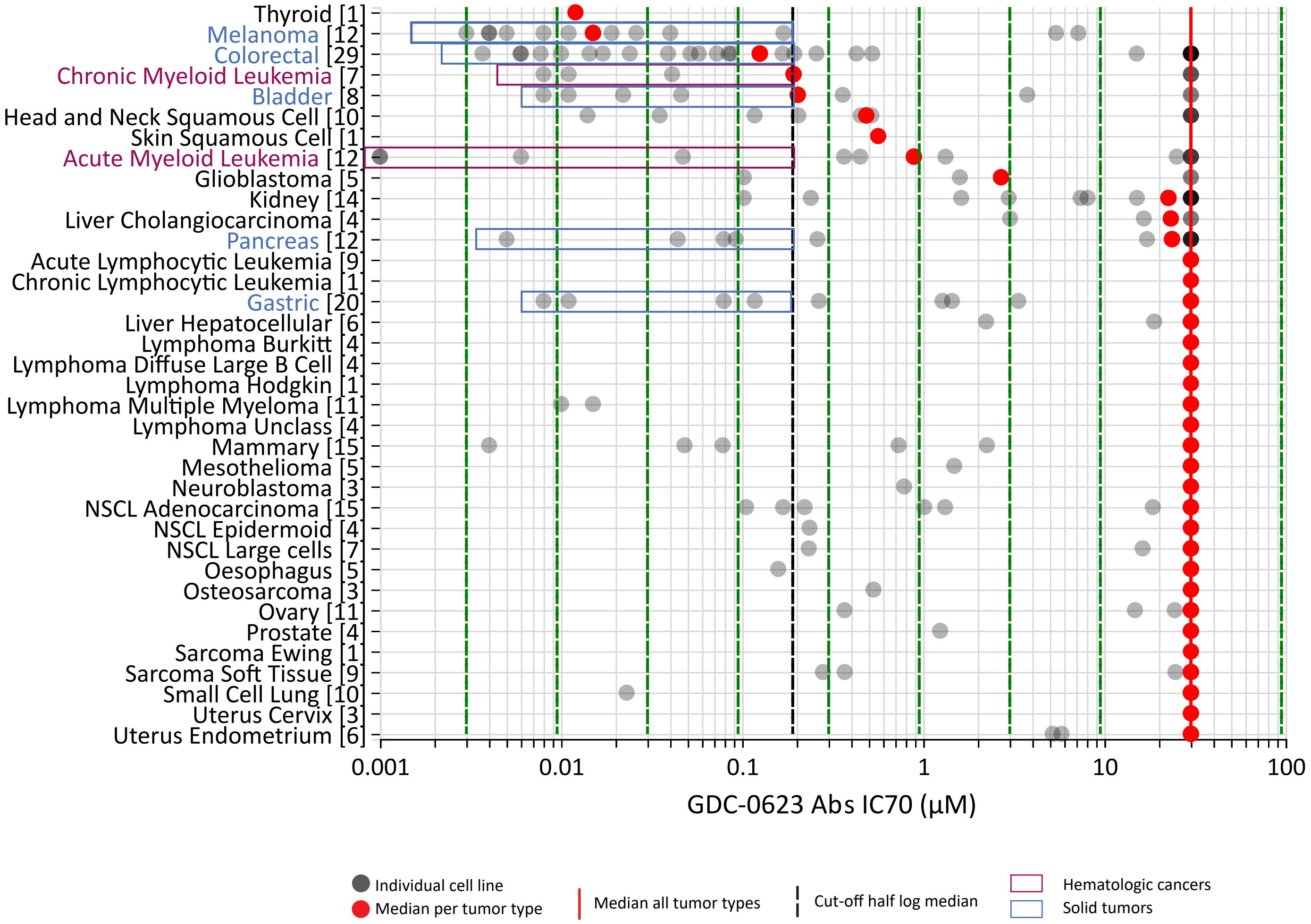

Supplementary Figure S1.
