# Supplementary material for: High In Vitro and In Vivo Activity of BI-847325, a Dual MEK/Aurora Kinase Inhibitor, in Human Solid and Hematologic Cancer Models
Source: Cancer Res Commun. 2023 Oct 25;3(10):2170–81. doi: 10.1158/2767-9764.CRC-22-0221 (PMC10599287; doi:10.1158/2767-9764.CRC-22-0221)
Supplement: Supplementary Figure S2 — shows the relative body weights of the mice treated with BI-847325 as monotherapy (first in vivo experiment). [file crc-22-0221-s03.pdf]

Supplementary materials

Supplementary Figure S2. In vivo effects of BI-847325 on mice relative body weight (from the screening series of experiments).

The relative body weight (%) over time (days) was used as an indicator of adverse effects. BI-847325 (80 or 40 mg/kg/day) or the vehicle control was administered orally on days 1, 8, 15, and 22. Black curves: control vehicle 10 ml/kg/day; light blue: BI-847325 at 40 mg/kg/day and dark blue: BI-847325 at 80 mg/kg/day; CXF 1103 and RKO: colorectal cancer; GXA 3011 and GXA 3023: gastric cancer; MAXFTN 401 and MDA-MB-231: triple-negative mammary cancer; MIA-PaCa-2: pancreatic cancer; RT112: bladder cancer.

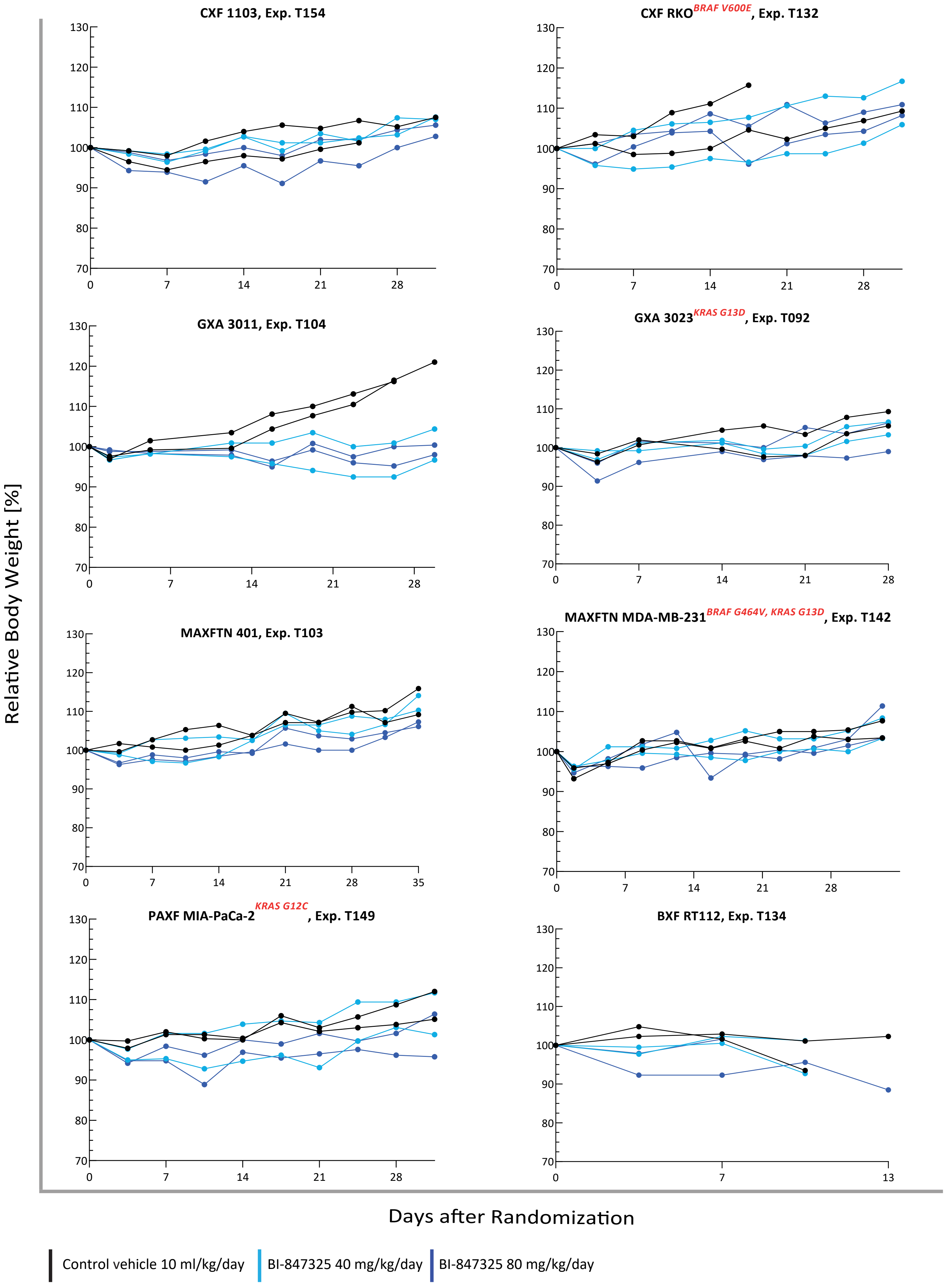

Supplementary Figure S2.
