# Supplementary material for: High In Vitro and In Vivo Activity of BI-847325, a Dual MEK/Aurora Kinase Inhibitor, in Human Solid and Hematologic Cancer Models
Source: Cancer Res Commun. 2023 Oct 25;3(10):2170–81. doi: 10.1158/2767-9764.CRC-22-0221 (PMC10599287; doi:10.1158/2767-9764.CRC-22-0221)
Supplement: Supplementary Figure S3 — shows the relative body weights of the mice treated with BI-847325 or capecitabine as monotherapy and in combination (second in vivo experiment). [file crc-22-0221-s04.pdf]

Supplementary materials

Supplementary Figure S3. In vivo effects of BI-847325 and capecitabine alone and in combination on mice relative body weight.

Black curves: Control vehicle 10 ml/kg/day on days 1, 8, and 15. Blue curves: BI-847325 administered orally at 80 mg/kg/day on days 1, 8, and 15. Green curves: capecitabine 150 mg/kg/day on days 1-7. Red curves: BI-847325 administered at 80 mg/kg/day on days 1, 8, and 15 (h:1) plus capecitabine administered at 150 mg/kg/day on days 1-7 (h:0). Purple curves: capecitabine administered at 150 mg/kg/day on days 1-7 plus BI-847325 at 80 mg/kg/day on days 8, 15, and 22. CXF 1103, colorectal cancer; GXA 3011 and GXA 3023, gastric cancer; MAXFTN 401, triple-negative mammary cancer.

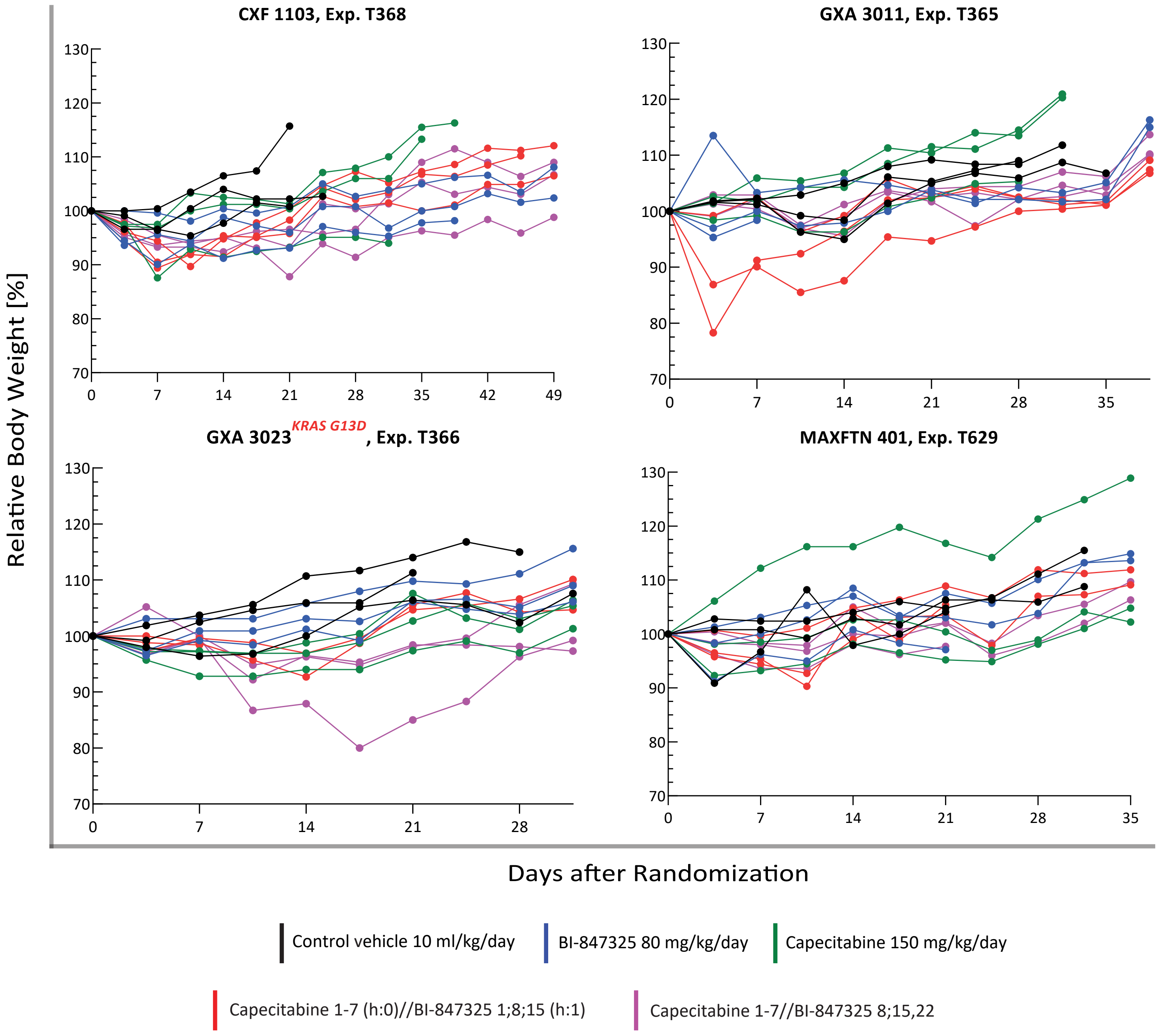

Supplementary Figure S3.
